# Neuroprotective effects of exercise in people with progressive multiple sclerosis (Exercise PRO-MS): study protocol of a phase II trial

**DOI:** 10.1186/s12883-020-01765-6

**Published:** 2020-05-11

**Authors:** A. S. Gravesteijn, H. Beckerman, B. A. de Jong, H. E. Hulst, V. de Groot

**Affiliations:** 1 Department of Rehabilitation Medicine, MS Center Amsterdam, Amsterdam Neuroscience research Institute, Amsterdam UMC, Vrije Universiteit Amsterdam, PO Box 7057, 1007 MB Amsterdam, the Netherlands; 2 Department of Neurology, MS Center Amsterdam, Amsterdam Neuroscience research Institute, Amsterdam UMC, Vrije Universiteit Amsterdam, PO Box 7057, 1007 MB Amsterdam, the Netherlands; 3 Department of Anatomy and Neurosciences, MS Center Amsterdam, Amsterdam Neuroscience research Institute, Amsterdam UMC, Vrije Universiteit Amsterdam, PO Box 7057, 1007 MB Amsterdam, the Netherlands

**Keywords:** Progressive multiple sclerosis, Exercise, High intensity interval training, Progressive resistance training, Neurodegeneration

## Abstract

**Background:**

Neurodegeneration, rather than inflammation, plays a key role in the progressive phase of multiple sclerosis (MS). Current disease modifying treatment options for people with progressive MS (PMS) do not specifically target neurodegeneration. Preliminary evidence suggests that exercise therapy might have neuroprotective effects. However, neuroprotective effect studies of exercise interventions in PMS are scarce and the possible mode of action underlying neuroprotective effects of exercise are unknown and need to be elucidated. The main aim of this phase II trial is to assess whether progressive resistance training (PRT) and high intensity interval training (HIIT), can slow down neurodegeneration in people with PMS.

**Methods:**

In a single-blinded phase II clinical trial with an extended baseline period, 60 people with PMS will be randomly assigned to PRT or HIIT. The participants should have had a relapse onset of MS with confirmed disease progression, however still ambulatory. The duration of the study is 48 weeks, consisting of 16 weeks baseline period (no intervention), 16 weeks intervention and 16 weeks follow-up. Patient-tailored training will be performed 3 times per week for one hour in groups, led by an experienced physiotherapist. The primary outcome measure is neurodegeneration, measured as whole brain atrophy on magnetic resonance imaging (MRI). Secondary outcome parameters will include other biomarkers associated with neurodegeneration (i.e. regional brain atrophy, lesion load, white matter integrity, resting state functional connectivity, blood biomarkers (brain derived neurotrophic factor (BDNF) and serum neurofilament light (sNFL)), patient functioning (physical and cognitive) and cardiovascular risk factors.

**Discussion:**

Besides the primary outcome measures, this study will examine a large variety of biomarkers associated with neurodegeneration after an exercise intervention. Combining outcome parameters may help to elucidate the mode of action underlying neuroprotective effects of exercise.

**Trial registration:**

This trial is prospectively registered at the Dutch Trial Registry (number NL8265, date 06-01-2020).

## Background

Multiple sclerosis (MS) is a complex disease of the central nervous system (CNS), with inflammatory and neurodegenerative aspects [1]. According to Lublin et al. MS can be categorized in different clinical subtypes, namely active versus not active neuroinflammation, with or without progression [2]. Progressive MS (PMS) is characterized by a gradual deterioration of daily activities due to increasing neurological symptoms [3]. The underlying pathophysiological mechanism for this deterioration is still poorly understood although it is known that neurodegeneration (i.e. axonal and neuronal loss in the CNS) plays a prominent role in PMS [1, 3]. Neurodegeneration is not yet targeted by the available disease modifying therapies (DMTs). These interventions generally target neuroinflammation [1, 3]. Some preliminary evidence from animal models, healthy aging individuals and people with MS suggests that exercise might have a neuroprotective effect [4–7].

### Exercise and neurodegeneration

Animal studies on the effects of exercise, demonstrated that endurance training in a mouse model of MS, i.e. experimental autoimmune encephalomyelitis (EAE), resulted in a better structurally preserved nervous system [4]. The endurance exercise group showed less severe motor impairment and autopsy showed less dendritic loss and less synaptic defects, compared to a sedentary mouse group [4].

Also, in healthy people research on the neuroprotective effects of exercise has been conducted. These studies suggest a neuroprotective effect of exercise and physical activity. Endurance training conducted at a moderate-to-vigorous intensity for six months resulted in an increase in grey- and white matter volume, especially in the prefrontal and temporal cortices [5]. Additionally, moderate intensity endurance training can upregulate levels of brain derived neurotrophic factor (BDNF) in healthy aging individuals [8, 9]. BDNF is a neurotrophin which plays an important role in synaptic plasticity, neuronal survival, differentiation and neuronal growth, and levels of BDNF decrease in neurodegenerative diseases [9–11]. Moreover, BDNF is strongly linked to cognitive functions [12, 13].

In people with MS exercise has been widely adopted as an intervention for patient functioning and general well-being. Exercise guidelines recommend twice weekly resistance training and endurance training to improve cardiorespiratory fitness, muscle strength, daily functioning and quality of life [14–16]. Cross-sectional functional MRI data indicate that people with MS who are more physically active (determined as averaged movement counts per day measured with an accelerometer) have increased resting state functional connectivity (rsFC) in the default-mode network (DMN) [17]. rsFC, measured with functional MRI, is a measure of the blood-oxygen-level-dependent signal used to examine the functional architecture of the brain [18]. The functional architecture of the brain in people with MS generally differs from that of healthy controls, and correlates with MS severity as measured by the Expanded Disability Status Scale (EDSS) [19]. In people with MS decreased rsFC of the DMN is associated with a decreased cognitive functioning [20, 21].

A recent systematic review indicated that 2–3 times exercise per week for at least 4 weeks can improve brain function and brain integrity in people with MS [6]. A 24-week progressive resistance training (PRT) intervention study in people with relapsing remitting MS, showed a trend towards brain preservation in the training group. A slowing of the natural loss of brain volume, expressed as percentage brain volume change (PBVC) was found of − 0.01%, (95% confidence interval (95%CI): − 0.15 to 0.13) compared to a PBVC of − 0.28, (95%CI: − 0.61 to 0.06) in the waitlist control group [7]. In addition, a better cardiorespiratory fitness is associated with a better integrity of the nervous system and larger (deep) grey matter brain volumes, except for the thalamus [22, 23]. Intervention studies in MS also examined the effect of exercise on rsFC. A 4-week intervention study combining endurance and resistance training demonstrated increased rsFC in the sensorimotor brain network areas [24]. The sensorimotor network plays an important role in motor function and rsFC in the sensorimotor network is decreased in people with MS [25]. Likewise, after a task-oriented walking intervention rsFC increased between sensorimotor areas [26]. An endurance walking intervention in people with MS demonstrated an improved rsFC between thalamic and right superior frontal gyrus, and thalamic and left medial frontal gyrus, associated with better cognitive performance [27]. In addition, white matter microstructural integrity measured with Diffusion Tensor Imaging (DTI) may improve with exercise [28]. Also in MS, an upregulation of BDNF after moderate intensity endurance training has been demonstrated [6, 11, 29, 30].

Exercise is a safe intervention for people with MS and current guidelines recommend exercise and physical activity to people with MS, because of the beneficial heath effect on patient functioning and cardiovascular risk [14, 31]. Exercise might be effective to reduce the cardiovascular risks associated with inactivity [15, 32]. The above-mentioned studies do indicate that exercise in people with MS has a positive effect on neurodegeneration. However, sample sizes in previous studies were small (N varying from 8 to 61), the content, frequency, intensity, and duration of exercise interventions differed, and outcome measures were heterogeneous (brain volume measures, rsFC, DTI-parameters, neuronal growth- and neurotrophic factors) [6]. Furthermore, there were no studies that examined the effect of exercise on neurodegeneration specifically in the relapse-onset PMS population. So, at this point the effects of different exercise interventions on neurodegeneration in the PMS population are not known.

### Exercise PRO-MS

In the proposed Exercise PRO-MS study an extensive set of neurodegenerative biomarkers will be used to investigate the effect of two patient-tailored exercise programs, i.e. PRT and high intensity interval training (HIIT) on neurodegeneration in people with PMS. Both resistance and endurance training might affect neurodegeneration but currently it is unknown whether one intervention is more beneficial than the other one. In the proposed study we will examine the effectiveness of both interventions on neurodegeneration in the PMS population.

The primary aim of this trial is:

To examine whether PRT and HIIT can slow down neurodegeneration in people with PMS. We expect that participants will benefit from both PRT and HIIT as compared to no training.

Additional objectives of the Exercise PRO-MS study are:

1. To determine whether PRT and HIIT can improve patient functioning in people with PMS.
2. To explore the relationships between patient functioning on the one hand, and biomarkers related to neurodegeneration on the other hand.
3. To determine the relative responsiveness of neurodegeneration outcome measures, and to explore effect sizes of between-intervention comparisons.
4. To determine whether cardiovascular risk factors improve as a consequence of a PRT or a HIIT intervention.

## Methods/design

### Design and setting

A single-blind monocenter randomized phase II trial with extended baseline will be conducted from 2020 till 2023 at the MS Center Amsterdam (MSCA), The Netherlands. MSCA is embedded in the Amsterdam UMC, location VUmc, and is a specialized center for MS care, research and education. In an extended baseline design, participants function as their own control reducing variance and increasing power. During intake participants will be assessed for eligibility for inclusion. In case of inclusion, participants will be scheduled for two baseline measurements sixteen weeks apart. After the second baseline measurement participants will be randomized to either the PRT or HIIT group. Measurements will be repeated post-intervention and after a 16-week follow-up period.

#### Randomization and blinding

Randomization will be performed to prevent selection bias. A computer-generated variable block-randomization model will be used. Block size will be disclosed at the end of the study. Block randomization is applied to achieve an approximately equal group size over time. An independent investigator will inform the physiotherapist about allocation of participants, participants will be notified of the intervention during the first training session. The outcome assessor will be unaware of allocation, and measures will be taken to ensure her blinding during the study. The physiotherapist and participant will not be blinded, since this is not possible.

### Study population

Participants will be recruited through the MS Center Amsterdam, patient organizations and social media. People who are interested will receive a patient information letter. If people are willing to participate an eligibility check will be planned. During this intake visit in- and exclusion criteria will be checked. Prior to all measurements participants will sign the informed consent form. If participants do not meet all inclusion criteria they will be excluded from the study. Inclusion criteria for this study are [1] definite diagnosis of MS according to the 2017 McDonalds criteria, with gradual progression of neurological impairments according to the Lublin criteria 2013 for at least 2 year [2, 33], [2] age between 18 and 70 years, [3] EDSS 3.5–6.0 [34, 35], [4] able to participate in exercise (i.e. no known cardiovascular, pulmonary or metabolic disease, or symptoms of cardiovascular disease), [5] able to understand instructions, and [6] fulfilling MRI safety criteria (no metal inside the body, no claustrophobia and no pregnancy). Exclusion criteria are [1] diagnosis of primary PMS (PPMS), [2] relapse within 3 months before baseline visit, [3] severe comorbidity, objectified as a score ≥ 3 in 1 or more organ systems on the Cumulative Illness Rating Scale (CIRS) [36], [4] initiation of fampridine within 6 months of baseline, [5] severe depression, objectified as a score ≥ 11 on the Hospital Anxiety and Depression Scale (HADS) [37], [6] other neurological- and/or musculoskeletal disorders, [7] already participating in a (guided) high intensity exercise training, [8] participating in another intervention study, and [9] pregnancy, given birth in previous 6 months or an active pregnancy wish.

All study procedures will be performed in accordance with the 1964 Helsinki Declaration and its later amendments or comparable ethical standards. Written informed consent will be obtained from all individual participants.

### Withdrawal and replacement of individual participants

**Fig. 1:**
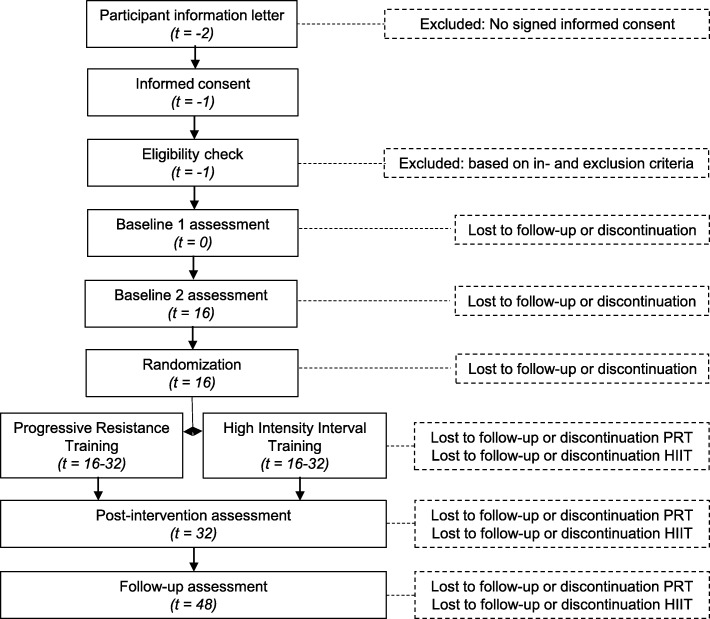
Participant Timeline

Participants can leave the study at any time for any reason if they wish to do so without any consequences. The investigator can decide to withdraw a participant from the study for urgent medical reasons. Participants who leave the study prior to random allocation to the exercise programs will be replaced. Participants who leave the study after random allocation will not be replaced. Participants that withdraw from the intervention will be asked to complete assessments post intervention and at follow-up. Also, they will be asked to share the reason for withdrawing from the study (Fig. 1).

### Sample size calculation

Based on pre- and post-intervention measurements, paired analysis will be performed to examine the effect of exercise per type of intervention. Power calculation was performed using data from a previous study examining the effect of a 24-week progressive resistance training (group difference of 0.3 PBVC, standard deviation (SD) 0.5, estimated effect size 0.6) [7]. A sample size of 24 achieves 80% power with a significance level of 0.05. With an estimated drop-out of 15% we will include 30 participants per group.

### Interventions

Both interventions (PRT & HIIT) will be performed three times per week for 16 weeks, supervised by a physiotherapist. Participants can start at different time points. So, group size and composition are variable. Training intensities are based on individual abilities. In case adjustments in exercises or intensities need to be made due to certain disabilities or circumstances, this will be documented and standardized.

#### Progressive resistance training (PRT)

The PRT schedule consists of 3 exercises for the lower extremity (leg press, unilateral leg extension and unilateral hamstring curl), 2 exercises for the upper extremity (bench press and seated rows) and 1 core exercise (cable wood chop), to improve muscle strength. Intensity will be at 60–80% of participants one-repetition maximum (1RM). The training will start with a 5-min warming-up on a bicycle ergometer followed by PRT (Table 1a). The training will end with a cooling down of 5 min on a bicycle ergometer and 5 min of stretching and relaxation (see Additional file 1 TIDieR Checklist PRT).

**Table 1:** Trainings programs, (A) progressive resistance training and (B) high intensity interval training

**A. Progressive Resistance Training**				**B. High Intensity Interval Training**				
*Week*	*Load (%1RM)*	*Volume*	*Rest (minutes)*	*Week*	*Sets*	*Interval (minutes)*	*Rest (minutes)*	*%peak heartrate*
1–2	60	3 × 12	2–3	1	5	1	1	80
				2	5	1	2	85
3–4	65	3 × 12	2–3	3	5	1	1	85
				4	5	2	2	80
5–6	70	3 × 10	2–3	5	5	2	2	85
				6	5	2	2	90
7	75	4 × 10	2–3	7	4	3	3	85
8	1RM measurement			8	Cardiopulmonary exercise test			
9–10	65	3 × 12	2–3	9	6	2	2	85
				10	4	3	3	80
11–12	70	3 × 10	2–3	11	4	3	3	85
				12	4	4	3	80
13–14	75	4 × 10	2–3	13	4	4	3	85
				14	4	4	3	90
15–16	80	4 × 10	2–3	15	5	2	2	95
				16	5	3	3	90

#### High intensity interval training

HIIT will be performed on a bicycle ergometer with high intensity interval and rest intervals alternated, to improve the cardiorespiratory fitness. The training will start with a 10-min warm-up on the bicycle on light-to-moderate intensity, then participants will start the HIIT training (Table 1b). The intervention will end with a cool down period of 5 min bicycling and 5 min of muscle stretching and relaxation (see Additional file 2 TIDieR Checklist).

During the intervention period and during the 16 weeks pre- and post-intervention participants are asked not to follow an exercise program outside this study, and to keep their level of care constant during this period when possible. Usual medical care is continued, including new treatments when the health situation of the patient changes.

#### Training intensity

For the PRT group, to determine muscle strength the 1RM of every exercise performed during the progressive resistance training will be determined in kg. For the HIIT group, cardiorespiratory fitness will be determined during a cardiopulmonary exercise test (CPET). Outcome measures are peak oxygen consumption (VO_2_peak) in oxygen per ml/min/kg, peak heartrate (HR peak) in beats per minute and peak load (Wpeak) in Watt. Muscle strength and cardiorespiratory fitness will be re-examined at week 8 of the intervention period, to optimize training intensity (Table 1).

### Outcome measures

Measurement will take place at baseline (week 0), extended baseline (week 16), post-intervention (week 32) and at follow-up (week 48) (Table 2).

**Table 2:** Schedule of enrollment and measurements

	**Enrollment**	**Baseline**		**Intervention period**	**Post-intervention**	
Week no:	-1	0	16	24	32	48
**Informed consent**	X					
**Screening**	X					
**Sociodemographic and clinical characteristics**		X				
**Neurodegeneration**						
Imaging		X	X		X	X
Blood sampling		X	X		X	X
**Patient functioning**						
• Mobility		X	X		X	X
• Dexterity		X	X		X	X
• Cognition		X	X		X	X
• Balance		X	X		X	X
• Disease		X	X		X	X
• Psychosocial		X	X		X	X
• Global perceived effect					X	X
**Cardiovascular risk factors**		X	X		X	X
**Activity level during follow-up**						X
**Training Intensity**						
1RM		X	X	X^a^	X	X
CPET		X	X	X^a^	X	X

^a^ Study group dependent

#### Primary outcome measure: percentage brain volume change

The primary outcome measure of this study is PBVC measured on MRI. A longitudinal assessment of whole brain atrophy will be determined using a high resolution T1-weighted image (i.e. three-dimensional magnetization-prepared rapid acquisition with gradient echo). Whole brain atrophy is an overall measure of neurodegeneration in MS [38, 39]. A valid software tool SIENA (part of FSL, https://fsl.fmrib.ox.ac.uk/fsl/fslwiki/SIENA) is available to determine PBVC automatically [40].

#### Other neurodegeneration biomarkers

##### Imaging

The following imaging markers will be determined:

- Grey matter, white matter, subcortical volumes and cortical thickness (as measured with respectively SIENA and FIRST (from fsl) and Freesurfer) [41].
- Lesion load using a fluid-attenuated inversion recovery (FLAIR) image [42].
- White matter integrity measured with DTI [41].
- rsFC will be calculated. In the proposed study we will focus on the rsFC in the sensorimotor network and DMN, which both can be determined reliably in people with MS [43]. Previous exercise intervention studies found changes in rsFC of the sensorimotor network after an exercise intervention [24, 26]. In older healthy individuals also changes in DMN are demonstrated [44, 45]. In people with MS the rsFC in the DMN is lower compared to healthy controls [20]. In physically active people with MS rsFC in the DMN is higher [17].

##### Blood sampling

Blood biomarkers including BDNF and serum Neurofilament Light (sNFL), will be assessed. Neurofilaments are the major component of axonal cytoskeleton proteins and during axonal damage, neurofilaments are released in the extracellular fluid [46]. sNFL gives an indication of the degree of axonal damage. Beside this, additional blood biomarkers will be determined. The field of research on MS and blood biomarkers is evolving rapidly. Based on these developments it will be determined in a later stage. To determine immunological responses to exercise immune cell profiles will be determined with a combination of flow cytometry by time of flight (CyTOF) mass cytometry and multi-parameter flow cytometry.

#### Patient functioning

##### Mobility

Different aspects of walking will be assessed. First, the energy cost of walking will be determined (6MWTec). 6MWTec is a measure of oxygen consumption per meter during walking, which is strongly associated with daily activity and perceived fatigue in MS [47]. Oxygen consumption will be determined during 6 min of walking with breath-by-breath gas exchange measurements. The last 3 min of the test are used to determine oxygen consumption, because steady state oxygen consumption is then assumed. Second, the 25-ft timed walk test (25 ft. TWT) will be performed. Participants will have to perform the 25 ft. TWT two times as fast as possible and two times on comfortable walking speed [48, 49]. Third, the patient-reported Multiple Sclerosis Walking Scale (MSWS-12), will be filled in [50].

##### Dexterity

Manual ability of both hands will be measured with the nine-hole peg test (9-HPT) [51].

##### Cognition

The Brief International Cognitive Assessment for MS (BICAMS) will be used to examine information processing speed and immediate verbal and visual recall [52]. The BICAMS consists of the Symbol Digit Modalities Test (SDMT), California Verbal Learning Test-II (CVLT-II) and the Brief Visuospatial Memory Test-Revised (BVMT-R). Parallel versions will be used to avoid learning effects. Fifteen healthy controls will also be included and followed over the same 48-week time span to be able to correct for learning effects in the analysis.

##### Balance

The Berg Balance Scale (BBS) will be used to assess balance [53, 54]. In this 14-item scale a rater scores different balance tasks, from sitting without support till standing on one leg, on a 0–4 scale. The total score will be between 0 and 56, a higher score representing better balance.

##### Disease severity and impact

A disease specific measurement of disability in MS is the EDSS combing impairment(s) in functional systems (pyramidal, cerebellar-, brain stem-, sensory-, bowel & bladder-, visual-, cerebral-, and other systems) and impairment in walking [34]. Disease impact will be measured with a 29-item patient-reported Multiple Sclerosis Impact Scale (MSIS-29) [55].

##### Psychosocial

Depression and anxiety will be assessed with the HADS [37, 56]. Resilience, the ability to bounce back from challenges occurring in life will be measured with the Connor Davidson Resilience Scale (CD-RISC25), a 25-item patient-reported outcome measure [57]. Fatigue will be determined using the Checklist Individual Strength-20r (CIS-20r), a patient-reported questionnaire [58, 59]. Quality of life will be assessed with the Short-Form health survey-36 (SF-36) [60, 61]. Exercise self-efficacy, confidence in performing exercise will be assessed with the EXercise Self-Efficacy scale (EXSE) [62, 63].

##### Domain specific perceived effect

The global changes over a specified time window will be measured using a single question scored on a 7-point scale, also known as the global perceived effect [64, 65]. Domain specific effect for muscle strength and cardiorespiratory fitness will be determined.

#### Cardiovascular risk

People with MS often have a worse cardiovascular risk profile compared to healthy individuals [66]. The unfavorable cardiovascular risk profile might be explained by a sedentary lifestyle common in MS [67]. In people with PMS the cardiovascular risk profile is even worse compared to people with relapse-remitting MS, but this might be explained by a further decline in mobility and daily functioning in the more progressive stages of the disease [68]. Exercise interventions might be beneficial for both patient functioning and cardiovascular risk factors [69, 70]. Studies do indicate that a higher cardiovascular risk is associated with higher lesion load, more brain atrophy and more disability progression [68, 71, 72]. The following cardiovascular risk factors will be determined; body weight, height, body mass index, % body fat, blood pressure, High-Density Lipoprotein (HDL), Low Density Lipoprotein (LDL), cholesterol, C-reactive Protein (CRP) and Hemoglobin A1c (HbA1c).

#### Demographic and clinical characteristics

Demographic and clinical characteristics will be determined at baseline. The following demographic characteristics will be determined: age, sex, educational level, level of physical activity with the International Physical Activity Questionnaire (IPAQ). The following clinical characteristics will be documented: comorbidities measured with CIRS, year of diagnosis, disease duration, use of medication, use of walking aid, level of education, work and hours of work per week.

#### Therapy adherence

Therapy adherence will be monitored. Presence or absence during training sessions will be documented. In the HIIT group the number of performed intervals and intensities will be documented and in the PRT group, the number of repetitions and sets will be documented as well as the weights.

### Statistical analysis

Statistical analysis will be performed using IBM SPSS (Chicago, Illinois) and/or R (Auckland, New Zealand). Nominal data will be presented as frequencies (n) and percentages (%). Ordinal and interval data will be presented as mean values (M) and SD in case of parametric data and median and range for non-parametric data. A *p*-value < .05 will be considered statistically significant for all analyses. To correct for multiple testing the Benjamini-Hochberg procedure will be used [73].

#### Descriptive statistics

Demographic and clinical characteristics such as age at first baseline assessment, sex, height, weight, %body fat, comorbidities with CIRS, medication, years since diagnosis, use of walking aid and EDSS score, will be summarized in a table. For therapy adherence and training intensity, frequencies and percentages will be calculated.

#### Primary objective

Within-group paired analysis will be performed for whole brain atrophy measured with PBVC at three different time intervals (difference between baseline 1 and 2, difference between baseline 2 and post- intervention and difference between post-intervention and follow-up) using linear mixed models The analysis will be performed separately for the two different exercise programs. The analysis will be performed using an intention-to-treat method, including all randomized participants, regardless of adherence and measurement completion. Secondary, per protocol analysis will be performed for further exploration of the intervention effects. In addition, one model for the total group of participants with the two different exercise programs as a determinant, will be explored.

#### Additional objectives

Longitudinal assessment of change in the different outcome measurements will be determined using a linear mixed model over four different time points. The relationship between (change in) patient functioning measures and (change in) neurodegeneration will be assessed using multiple regression analyses. The relative responsiveness of the various neurodegenerative biomarkers will be analyzed by relating the smallest real difference to the minimally important change of the outcome measures and comparing the area under the receiver operation characteristics curves [74]. Domain-specific patient perceived effect parameters will be used as an anchor. To analyze the effects on cognitive performance, a Reliable Change Index (RCI) will be calculated based on the mean and standard deviation of the healthy controls to adjust for learning effects [75]. An RCI of ±1.64 (90%) will be considered a significant improvement/decline.

### Study status

Currently, participants are being recruited (May 2020).

## Discussion

In PMS neurodegeneration plays a key role in the progression of disability. Up to date there are no DMTs affecting the non-inflammatory drivers of the disease. Previous studies in animals, healthy aging people, and people with MS indicate that exercise training might result in neuroprotective effects and reduced neurodegeneration. However, the interventions used varied, study groups were heterogeneous and sample sizes were most often too small. In this study, the effect of two exercise interventions, PRT and HIIT, will be specifically examined in a homogeneous cohort of people with PMS. Both training interventions are expected to induce neuroprotective effects. Currently it is not known which intervention is more beneficial. In this trial a large variety of neuroprotective outcome measures will be assessed, such as structural and functional imaging parameters of the brain but also biomarkers in blood. In addition, several patient functioning measures will be assessed, for example mobility, dexterity and cognition. Also, the preventive effect of exercise on patient’s cardiovascular risk will be studied. The combination of all these outcome measures is one of the major strengths of this study and will hopefully give better insight in the neuroprotective effect of high intensity exercise programs in people with PMS.

In the proposed study we will examine the biomarkers BDNF and sNFL. However, a lot of ongoing research is examining new possible biomarkers for PMS. For example, Glial Fibrillary Acid Protein (GFAP), Vascular Endothelial Growth Factor (VEGF) and sContactin1 and 2. GFAP is a major intermediate cytoskeletal protein of astrocytes and an increase in levels of GFAP is associated with more astrogliosis [76, 77]. VEGF plays a key role in angiogenesis and seems to play a role in neurogenesis [78, 79]. sContactin1 and sContactin2 are potential biomarkers for axonal dysfunction [80]. However, the research on biomarker development is still ongoing and future research might unveil other, more specific neurodegenerative biomarkers. Therefore, participants will be asked to donate some extra blood for possible future analyses.

## Supplementary information

**Additional file 1.** A document containing the TIDieR checklist for the PRT Intervention.

**Additional file 2.** A document containing the TIDieR checklist for the HIIT intervention.

## Data Availability

Data sharing is not applicable to this article as no datasets were generated or analyzed during the current study. Datasets (generated and/or analyzed) during the proposed study will be available from the corresponding author on reasonable request.

## Abbreviations

1RM: 1-repitition maximum
25 ft. TWT: 25-ft timed walk test
6MWTec: 6-min walk test with energy cost measurement
95%CI: 95% confidence interval
AE: Adverse event
BBS: Berg Balance Scale
BDNF: Brain derived neurotropic factor
BICAMS: Brief international cognitive assessment for multiple sclerosis
BVMT-R: Brief visuospatial memory test-revised
CD-RISC 25: Connor Davidson resilience scale
CIRS: Cumulative illness rating scale
CIS 20r: Checklist individual strength 20r
CNS: Central nervous system
CPET: Cardiopulmonary exercise test
CRB: Clinical research bureau
CRP: C-reactive protein
CVLT-II: California verbal learning test-II
CyTOF: Cytometry by time of flight
DMN: Default-mode network
DMTs: Disease modifying therapies
DTI: Diffusion tensor imaging
EAE: Experimental autoimmune encephalomyelitis
EDSS: Expanded disability status scale
EXSE: Exercise self-efficacy scale
FLAIR: Fluid-attenuated inversion recovery
GCP: Good clinical practice
GFAP: Glial fibrillary acid protein
HADS: Hospital anxiety and depression scale
HbA1c: Hemoglobin A1c
HDL: High-density lipoprotein
HIIT: High intensity interval training
HR peak: Peak heartrate
IPAQ: International physical activity questionnaire
LDL: Low-density lipoprotein
METc: Medical ethical committee
MS: Multiple sclerosis
MSCA: Multiple sclerosis center Amsterdam
MSIS-29: Multiple sclerosis impact scale-29
MSWS− 12: Multiple sclerosis walking scale-12
NHPT: Nine-hole peg test
PBVC: Percentage brain volume change
PMS: Progressive multiple sclerosis
PPMS: Primary progressive multiple sclerosis
PRT: Progressive resistance training
RCI: Reliable change index
rsFC: Resting state functional connectivity
SAE: Serious adverse event
SD: Standard deviation
SDMT: Symbol digit modalities test
SF-36: Short -form health survey-36
sNFL: Serum neurofilament light
VEGF: Vascular endothelial growth factors
VO_2_peak: Peak oxygen consumption
Wpeak: Peak load in watt
