# Supplementary material for: Neuroprotective effects of exercise in people with progressive multiple sclerosis (Exercise PRO-MS): study protocol of a phase II trial
Source: BMC Neurol. 2020 May 11;20:177. doi: 10.1186/s12883-020-01765-6 (PMC7212565; doi:10.1186/s12883-020-01765-6)
Supplement: Supplementary file 1 — Additional file 1. A document containing the TIDieR checklist for the PRT Intervention. [file 12883_2020_1765_MOESM1_ESM.docx]

Brief Name: Progressive Resistance Training in progressive multiple Sclerosis

TIDieR Checklist

# Why

This interventions is part of the Exercise PRO-MS trial on the neuroprotective effects of exercise training in patients with progressive multiple sclerosis. Multiple Sclerosis guidelines prescribe resistance training for at least two times per week (1). Resistance training is beneficial for muscle strength, fatigue, quality of life and functional capacity in people with Multiple Sclerosis (2,3). In addition, a randomized controlled trial demonstrated that progressive resistance training (PRT) resulted in a trend towards brain preservation in people with multiple sclerosis (4).

# What materials

Resistance training machines: Leg press, Leg extension, Leg press, pully, bench, barbel and a power rack.

Informational materials: a form with the different exercises will be provided for the participants (the Dutch version of table 1) and a training schedule will be printed for every participant.

# What procedures

The PRT focusses on large muscle groups of upper and lower extremities and core. Exercises are: leg press, unilateral leg extension, unilateral hamstring curl, bench press, upright row and cable wood chop. The training will start with a 5-minute warm-up phase on a bicycle ergometer. The training will end with a cool down session consisting of 5 minutes on the bicycle ergometer and 5 minutes of stretching. Intensity of the exercises will be based on a test of the 1-repetition maximum (1RM) per exercise, except for the cable wood chop exercise (3).

**Table 1: Different exercises**

| **Exercises Lower Extremity** | |
| --- | --- |
| 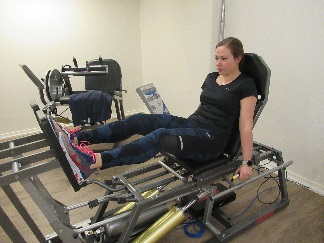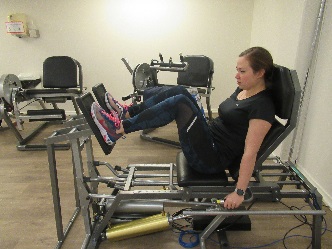 | **Leg press**  Start in 90 degrees bend position of the knees and hips, quick to near maximal extension and slowly return to the starting position. |
| 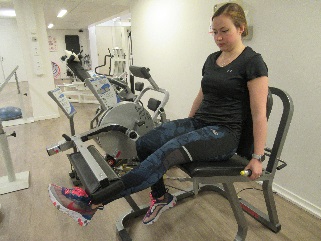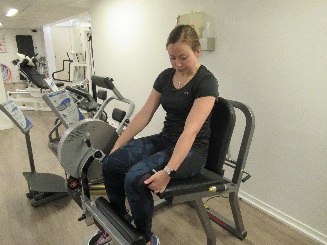 | **Unilateral leg extension:**  Start in 90 degrees bend position of the knees and hips. Quickly extend one knee and then slowly return to the starting position. |
| 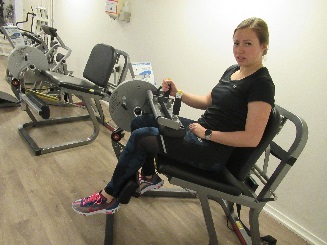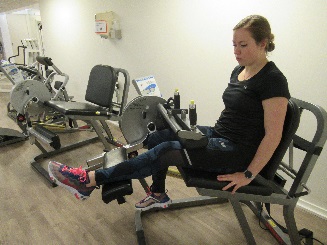 | **Unilateral hamstring curl:**  Start with near to extended knee position and quickly flex knees to a 90 degrees position, then slowly return to the start position. |
| **Exercises Upper Extremity** | |
| 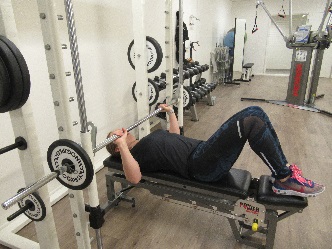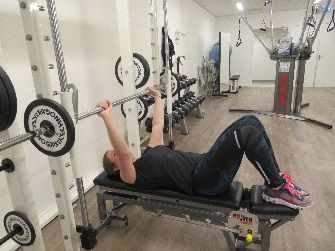 | **Bench Press:**  Lift weight out of stand and start with extended arms, slowly flex arms and bring barbell towards chest, extend arms quickly and exhale at the same time. |
| 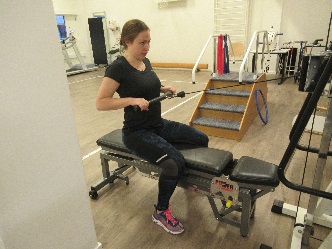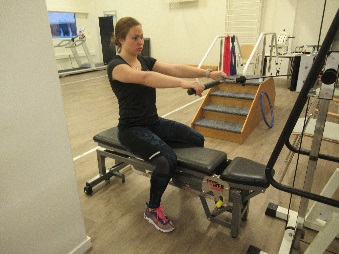 | **Seated Row:**  Start with extended arms in an upright position. The pulley is already somewhat pulled out. Flex arms and stay in upright position then slowly return to starting position. |
| **Core Exercise** | |
| **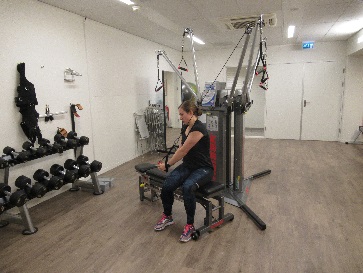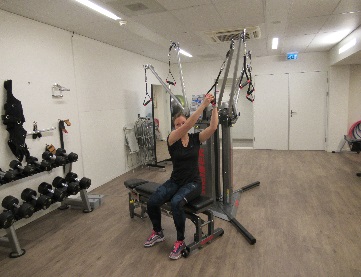** | **Two-sided Cable wood chop:**  Start in the upper corner and pull down toward the opposite site towards the hip. |

# Who provided

The progressive resistance training is supervised by an experienced physiotherapist. The physiotherapist is additionally trained by the executive investigator in the specific exercise program requirements for this study.

# How

Training will be performed in groups of maximally 5 persons under direct supervision of the physiotherapist. Instructions will be given face-to-face. In addition, participants have their own training schedule with basic instructions.

# Where

The intervention will be performed at the fitness hall of the department of rehabilitation medicine at Amsterdam UMC, location VUmc.

# When and How Much

During a period of 16 weeks participants will train three times per week for one hour. The training intensities and dosage are presented in table 2.

**Table 2 : 16-week Progressive Resistance Training; Progression scheme:**

| **week** | **Load** | **Volume** | **Rest** |
| --- | --- | --- | --- |
| 1-2 | 60% 1RM | 3 sets 12 repetitions | 2-3 minutes |
| 3-4 | 65% 1RM | 3 sets 12 repetitions | 2-3 minutes |
| 5-6 | 70% 1RM | 3 sets 10 repetitions | 2-3 minutes |
| 7 | 75% 1RM | 4 sets 10 repetitions | 2-3 minutes |
| 8 | 1 RM measurement | | |
| 9-10 | 65% 1RM | 3 sets 12 repetitions | 2-3 minutes |
| 11-12 | 70% 1RM | 3 sets 10 repetitions | 2-3 minutes |
| 13-14 | 75% 1RM | 4 sets 10 repetitions | 2-3 minutes |
| 15-16 | 80% 1RM | 4 sets 10 repetitions | 2-3 minutes |

# Tailoring

The intensity of the exercises will be patient-tailored, based on 1RM. The cable wood chop can be performed in a standing or sitting position depending on balance. If participants are not able to perform a specific exercise exercises will be modified to the participant’s ability. Modifications will be documented in a patient log. Documentation will consist of reason for modification, type of modification and when modifications are made.

# Modifications

Not applicable

# How well (planned)

Every training session will be recorded in a patient training diary. In this document participants will makes notes of the number of completed sets, repetitions and load. In addition participants will document the perceived exertion.

# How well (actual)

Not applicable

# References

1. Latimer-Cheung AE, Martin Ginis KA, Hicks AL, Motl RW, Pilutti LA, Duggan M, et al. Development of evidence-informed physical activity guidelines for adults with multiple sclerosis. Arch Phys Med Rehabil. 2013;94(9):1829-1836.e7.

2. Cruickshank TM, Reyes AR, Ziman MR. A systematic review and meta-analysis of strength training in individuals with multiple sclerosis or parkinson disease. Med (United States). 2015;94(4):1–15.

3. Dalgas U, Stenager E, Jakobsen J, Petersen T, Hansen HJ, Knudsen C, et al. Resistance training improves muscle strength and functional capacity in multiple sclerosis. Neurology. 2009;73(18):1478–84.

4. Kjølhede T, Siemonsen S, Wenzel D, Stellmann J-P, Ringgaard S, Pedersen BG, et al. Can resistance training impact MRI outcomes in relapsing-remitting multiple sclerosis? Mult Scler J. 2018 Sep 28;24(10):1356–65.
