# Supplementary material for: Neuroprotective effects of exercise in people with progressive multiple sclerosis (Exercise PRO-MS): study protocol of a phase II trial
Source: BMC Neurol. 2020 May 11;20:177. doi: 10.1186/s12883-020-01765-6 (PMC7212565; doi:10.1186/s12883-020-01765-6)
Supplement: Supplementary file 2 — Additional file 2. A document containing the TIDieR checklist for the HIIT intervention. [file 12883_2020_1765_MOESM2_ESM.docx]

Brief Name: High Intensity Interval Training in Progressive Multiple Sclerosis

TIDieR Checklist

# Why

This intervention is part of the Exercise PRO-MS trial on the neuroprotective effects of exercise training in patients with progressive multiple sclerosis. Multiple Sclerosis guidelines prescribe endurance training for at least two times per week (1). Endurance training is beneficial for cardiorespiratory fitness, mobility, fatigue and quality of life in people with MS (2). An intervention study assessing the effect of endurance training in healthy aging individuals demonstrated an increase in grey and white matter volume (3). In people with MS it is already demonstrated that after a moderate intensity endurance intervention people with MS have increased levels of brain derived neurotrophic factor (4). Performing endurance training at higher intensity, such as high intensity interval training (HIIT), is expected to result in more neuroprotective effects in people with MS (5). A study comparing HIIT with moderate intensity endurance training demonstrated that the HIIT group improved more in cardiorespiratory fitness than the moderate intensity interval training group (6).

# What materials

The HIIT intervention will be performed on a bicycle ergometer. All participants will receive a printed version of the training schedule.

# What procedures

During a 16-week training period, HIIT will be performed on a bicycle ergometer. The training will start with a 10-minute warming-up period at preferred intensity on the ergometer. The training will end with a cool down session consisting of 5 minutes on preferred load on the bicycle ergometer and 5 minutes of stretching. Intensity of the interval training will be based on the outcome of a cardiopulmonary exercise test (CPET).

# Who provided

The high intensity interval training is supervised by an experienced physiotherapist. The physiotherapist is additionally trained by the executive investigator in the specific exercise program requirements for this study.

# How

Training will be performed in groups of maximally 5 persons under direct supervision of the physiotherapist. Instructions will be given face-to-face. In addition, participants have their own training schedule with basic instructions.

# Where

The intervention will be performed at the fitness hall of the department of rehabilitation medicine at Amsterdam UMC, location VUmc, The Netherlands.

# When and How Much

During a period of 16 weeks participants will train three times per week for one hour. The training intensities and dosage are presented in table 1.

**Table 1 HIIT 16-week program; progression scheme :**

| **week** | **sets** | **Interval** | **Rest** | **% peak Heartrate** |
| --- | --- | --- | --- | --- |
| 1 | 5 | 1 min | 1 min | 80 |
| 2 | 5 | 1 min | 2 min | 85 |
| 3 | 5 | 1 min | 1 min | 85 |
| 4 | 5 | 2 min | 2 min | 80 |
| 5 | 5 | 2 min | 2 min | 85 |
| 6 | 5 | 2 min | 2 min | 90 |
| 7 | 4 | 3 min | 3 min | 85 |
| 8 | CPET to update training intensity | | | |
| 9 | 6 | 2 min | 2 min | 85 |
| 10 | 4 | 3 min | 3 min | 80 |
| 11 | 4 | 3 min | 3 min | 85 |
| 12 | 4 | 4 min | 3 min | 80 |
| 13 | 4 | 4 min | 3 min | 85 |
| 14 | 4 | 4 min | 3 min | 90 |
| 15 | 5 | 2 min | 2 min | 95 |
| 16 | 5 | 3 min | 3 min | 90 |

# Tailoring

The intensity of the exercises will be patient-tailored, based on CPET results, especially the peak heartrate. HIIT will be performed on a bicycle ergometer for safety reasons (i.e. lower fall risk). In case modifications need to be made this will be documented, in a patient log. Documentation will consist of date of modifications, type of modification and why they are made.

# Modifications

Not applicable

# How well (planned)

Presence or absence during training sessions will be documented. Every training session will be recorded in a patient training diary. In this document participants will describe the number of completed intervals, load and heartrate. In addition, participants will document the perceived exertion.

# How well (actual)

Not applicable

# References

1. Latimer-Cheung AE, Martin Ginis KA, Hicks AL, Motl RW, Pilutti LA, Duggan M, et al. Development of evidence-informed physical activity guidelines for adults with multiple sclerosis. Arch Phys Med Rehabil. 2013;94(9):1829-1836.e7.

2. Feys P, Moumdjian L, Van Halewyck F, Wens I, Eijnde BO, Van Wijmeersch B, et al. Effects of an individual 12-week community-located “start-to-run” program on physical capacity, walking, fatigue, cognitive function, brain volumes, and structures in persons with multiple sclerosis. Mult Scler J. 2019;25(1):92–103.

3. Colcombe SJ, Erickson KI, Scalf PE, Kim JS, Prakash R, McAuley E, et al. Aerobic Exercise Training Increases Brain Volume in Aging Humans. Journals Gerontol Ser A Biol Sci Med Sci. 2006 Nov 1;61(11):1166–70.

4. Negaresh R, Motl RW, Zimmer P, Mokhtarzade M, Baker JS. Effects of exercise training on multiple sclerosis biomarkers of central nervous system and disease status: a systematic review of intervention studies. Eur J Neurol. 2019 May 14;26(5):711–21.

5. Collett J, Dawes H, Meaney A, Sackley C, Barker K, Wade D, et al. Exercise for multiple sclerosis: A single-blind randomized trial comparing three exercise intensities. Mult Scler J. 2011;17(5):594–603.

6. Zimmer P, Bloch W, Schenk A, Oberste M, Riedel S, Kool J, et al. High-intensity interval exercise improves cognitive performance and reduces matrix metalloproteinases-2 serum levels in persons with multiple sclerosis: A randomized controlled trial. Mult Scler J. 2018 Oct 21;24(12):1635–44.
